# Effectiveness of cricoid pressure in preventing gastric aspiration during rapid sequence intubation in the emergency department: study protocol for a randomised controlled trial

**DOI:** 10.1186/1745-6215-13-17

**Published:** 2012-02-16

**Authors:** Christopher E Trethewy, Julie M Burrows, Don Clausen, Steven R Doherty

**Affiliations:** 1 Tamworth Rural Referral Hospital, Tamworth, New South Wales, Australia; 2 University Department of Rural Health and Rural Clinical School, University of Newcastle, Tamworth, New South Wales, Australia; 3 Pathology New England, Tamworth, New South Wales, Australia

**Keywords:** Rapid Sequence Intubation (RSI), cricoid pressure, aspiration, pepsin

## Abstract

**Background:**

Cricoid pressure is considered to be the gold standard means of preventing aspiration of gastric content during Rapid Sequence Intubation (RSI). Its effectiveness has only been demonstrated in cadaveric studies and case reports. No randomised controlled trials comparing the incidence of gastric aspiration following emergent RSI, with or without cricoid pressure, have been performed. If improperly applied, cricoid pressure increases risk to the patient. The clinical significance of aspiration in the emergency department is unknown. This randomised controlled trial aims to; 1. Compare the application of the 'ideal" amount of force (30 - 40 newtons) to standard, unmeasured cricoid pressure and 2. Determine the incidence of clinically defined aspiration syndromes following RSI using a fibrinogen degradation assay previously described.

**Methods/design:**

212 patients requiring emergency intubation will be randomly allocated to either control (unmeasured cricoid pressure) or intervention groups (30 - 40 newtons cricoid pressure). The primary outcome is the rate of aspiration of gastric contents (determined by pepsin detection in the oropharyngeal/tracheal aspirates or treatment for aspiration pneumonitis up to 28 days post-intubation). Secondary outcomes are; correlation between aspiration and lowest pre-intubation Glasgow Coma Score, the relationship between detection of pepsin in trachea and development of aspiration syndromes, complications associated with intubation and grade of the view on direct largyngoscopy.

**Discussion:**

The benefits and risks of cricoid pressure application will be scrutinised by comparison of the incidence of aspiration and difficult or failed intubations in each group. The role of cricoid pressure in RSI in the emergency department and the use of a pepsin detection as a predictor of clinical aspiration will be evaluated.

**Trial registration:**

Australian New Zealand Clinical Trials Registry (ANZCTR): ACTRN12611000587909

## Background

Rapid sequence intubation (RSI) is the process by which anaesthetic and neuromuscular blocking agents are administered in rapid succession to facilitate endotracheal intubation [1]. In the emergency department (ED) RSI is typically performed on acutely ill or injured patients who are often not fasted, increasing the risk of gastric aspiration during the process. In 1961 Sellick reported using "occlusion of the upper oesophagus by backwards pressure on the cricoid ring against the bodies of the cervical vertebrae to prevent gastric contents from reaching the pharynx" during induction of anaesthesia in patients at high risk of aspiration [2]. Cricoid pressure (CP) was subsequently adopted as an integral component of RSI in EDs despite inadequate scientific evaluation of its risks and benefits; its effectiveness has subsequently only been demonstrated in cadavers [3-6]. No randomised controlled trials have shown any benefit of its use during RSI. Furthermore the application of CP may be associated with increased risks to the patient: impeding airway management [7,8]; prolonging intubation time by obscuring laryngeal view [9,10]; inducing nausea/vomiting [10] and oesophageal rupture with excessive force [11]. Paradoxically, CP may indeed promote aspiration by relaxing the lower part of the oesophagus [12]. Some case reports note that tracheal intubation was impeded by CP [13] and regurgitation occurred despite application of CP [14,15], possibly due to its improper application.

Ostensibly a simple procedure, CP is in practice a complex manoeuvre which is difficult to perform optimally [12]. The amount of force used by the cricoid operator is a crucial factor in proper application of CP. A force in the range of 30 - 40 Newtons (N) applied to the cricoid ring is generally accepted as sufficient to occlude the oesophagus [5,12,16-18], preventing regurgitation of gastric contents. Studies have revealed that the knowledge of cricoid force theory and concept of the forces required is poor amongst both anaesthetic and ED staff [19-31]. Considerable ranges in the amount of force used by staff when applying force to cricoid models have been described [29,31,32]. Furthermore, when ED staff were asked to apply "ideal" CP to a human larynx model, only 25% of the group reached the target range, while 47% applied insufficient and 28% applied excessive force [29]. At forces greater than 44 N the patient is considered to be at risk of oesophageal rupture [5,10]. Training staff how to gauge the correct amount of force to use is effective in the short term only, however, in practice training is rarely undertaken [23,25,26,31,33,34]. In addition, the effect of fatigue on the ability of the cricoid operator to maintain CP within the target range for the duration of prolonged intubations has been questioned [35]. The use of floor scales to provide a means of gauging the amount of force applied to a cricoid model has been demonstrated [25], however its use in real-time measurement during RSI has not been reported.

Aspiration of gastric or oropharyngeal contents into the larynx and lower respiratory tract may lead to various pulmonary syndromes, most notably pneumonia and pneumonitis. The true incidence and clinical significance of aspiration during RSI is difficult to establish due to the heterogeneous nature of pathologic processes of critically ill patients requiring an emergency airway. Compounding this is the fact that many patients aspirate prior to intubation or arrival at ED and that repeated attempts at intubation increased the incidence of aspiration (Mort et al. 2004). Mort reported that the rate of aspiration increased from 1.9% with one attempt at intubation to 22% with three or more attempts [36]. Given these limitations, the incidence of aspiration appears to lie anywhere between 0.8% [36] and 22% [37]. There is currently no widely available test to determine whether pulmonary aspiration has occurred during RSI. Pepsin detection has been described as a potential aspiration biomarker [38] since it is uniquely secreted in stomach. Ufberg has developed and validated a specific and sensitive pepsin assay as a marker of aspiration [39]. Using the assay he found pepsin in the lungs in 50% of patients intubated in the pre-hospital setting, and 22% of patients undergoing RSI in the ED [37], however there was no longitudinal follow-up to see whether the presence of pepsin in the lungs was linked to subsequent development of aspiration syndromes. It was also not possible to determine if aspiration occurred prior to or at the time of intubation.

The value of CP in RSI is under increasing scrutiny [40,41]. Since its introduction in the 1960s anaesthesia practices and airway management techniques have improved leading some to wonder what contribution CP now makes towards reduced aspiration rates [40]. Critically ill patients presenting to the ED are also likely to have aspirated prior to intubation [40], therefore negating necessity for CP in the ED.

Emergency physicians today need evidence to support the use of CP in the ED. The current study aims to address these issues, being designed to evaluate the effectiveness, safety and need for CP during RSI in EDs.

### Study design

This is a prospective randomised blinded controlled study of RSI and CP application within the ED. Due to ethical considerations, a control group without CP applied cannot be included in any study. Instead, this study will exploit the fact that the target range CP of 30 - 40 newtons (N) was applied only 25% of the time by ED staff when tested on a model larynx [29]. The incidence of aspiration (determined by pepsin detection in the trachea) in two groups of patients will be compared; those with conventional CP (and hence about a 25% chance of target range CP), and those with exact sustained target range CP (using weighing scales for direct biofeedback). Longitudinal follow-up of participants will be performed to determine to what degree detection of pepsin in the trachea correlates with the development of clinical aspiration syndromes. In addition the incidence of aspiration as a result of pre-hospital intubation (determined by pepsin detection in the trachea) will be determined.

### Study objectives

#### Primary aims

• Test the hypothesis that the use of CP during RSI in the ED does not prevent aspiration.

• Investigate the effect of CP on RSI and associated incidence of difficulty or failed intubation.

• Determine the incidence of pre-hospital aspiration.

#### Secondary aims

• Determine whether a relationship exists between the incidence of aspiration and pre-intubation Glasgow coma score (GCS).

• Determine any correlation between pepsin detection in aspirates and development of clinical aspiration syndromes.

### Setting

Three hospitals will participate in the trial: Tamworth Rural Referral Hospital (Hunter New England Local Health District), and Port Macquarie and Coffs Harbour Base Hospitals (Mid North Coast Local Health District).

### Participants

Adult patients (> 18 years) undergoing RSI in the ED will be invited to participate in the study. Patients will be randomised to receive either standard CP (Control arm) or sustained "ideal" CP (Intervention arm). Patients will be excluded from the trial if cardiopulmonary resuscitation (CPR) is in progress upon arrival at the ED.

### Consent

Consent will be sought from the patient (or their Next of Kin) to participate in the trial, for testing the endotracheal and oropharyngeal aspirates for pepsin and for the 28 day follow-up. The right to refuse is clearly stated on the consent form. Patient consent is sought by the CP operator. In instances where a relative consents for the trial, participant consent is sought as soon as practicable after they have been successfully intubated and stabilised.

The study protocol has been approved by both the Hunter New England Human Research Ethics and the North Coast Area Health Service Human Research Ethics Committees.

### Randomisation

Patients will be randomised to the treatment groups by a directive included in the enrollment pack which is not apparent until the pack is opened to ensure all staff members are blinded to the participant's group allocation.

### Interventions

#### Intervention arm

The cricoid operator will stand on a set of scales with a visible display providing direct feedback of the force applied to the cricoid while performing CP. When required, the operator will apply 30 - 40 N force (equating to a weight displacement of 3.060 - 4.075 kg [29]) to the cricoid during RSI unless otherwise instructed by the intubation operator. Force will be applied until the endotracheal tube (ETT) placement is confirmed by end tidal CO_2 _and the ETT cuff is inflated.

#### Control arm

The cricoid operator will stand on a set of scales while performing CP. The scale display will be covered to blind the operator to the amount of force they are using. When required, the cricoid operator will apply what they consider to be 30 - 40 N force to the cricoid during RSI unless otherwise instructed by the intubation operator. Force will be applied until the ETT placement is confirmed by end tidal CO_2 _and the ETT cuff is inflated.

### Outcome measures

#### Primary outcome measures

• Rate of aspiration (pepsin detection in tracheal aspirate)

• Treatment for aspiration pneumonitis

#### Secondary outcome measures

• Grade of laryngeal view

• Detection of pepsin in oropharyngeal aspirates

• Procedural complications associated with intubation

• Immediate complications (associated with RSI) within 30 minutes of intubation

• Lowest pre-intubation GCS

• Survival to 28 days/hospital discharge

### Methods

#### Cricoid pressure measurement

**Figure 1:**
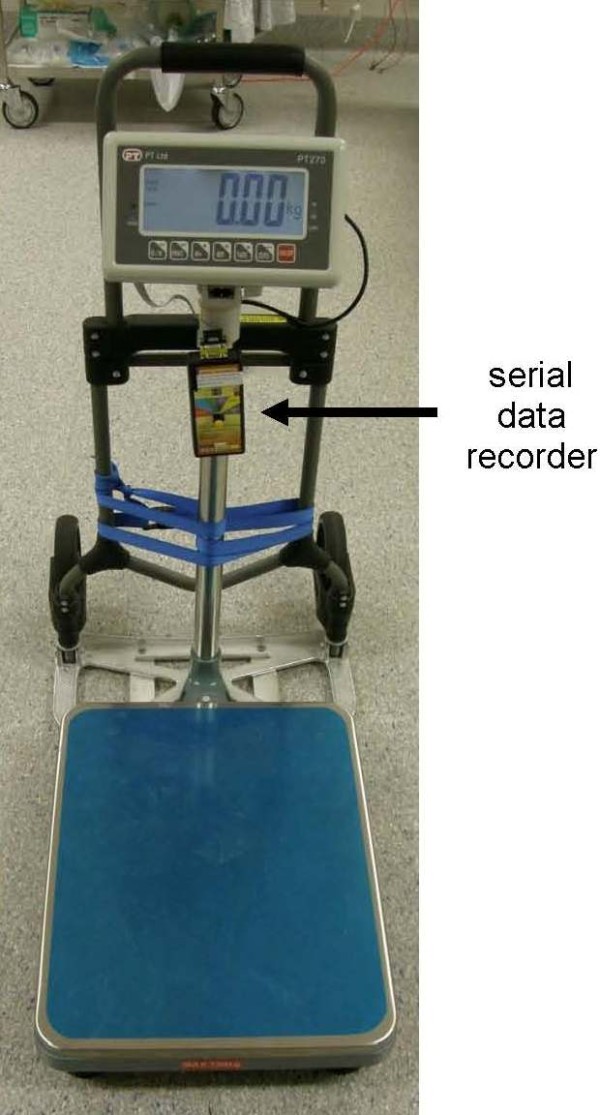
**Platform scale with pole mounted display and serial data logger connected, secured to a hand trolley**.

A platform scale with pole-mounted liquid crystal display LCD (150 kg capacity) set to record in 0.01 kg increments is used to measure cricoid pressure (Figure 1. Model PT270, PT Global Pty Ltd., NSW, Australia). The scale is mounted on a hand trolley with a custom made extended toe plate (designed to support the base plate of the scale without interfering with its levelling feet) allowing it to be easily manoeuvred into position next to the resus bed when required. Once in position and turned on, the cricoid operator stands on the scale and select the tare option to display their body weight as zero. Direct feedback of the force applied to the cricoid is available via the displayed weight reduction (a reading of -1 kg equates to approximately 10 N force). The cricoid operator maintains contact only with the scale baseplate and the patient's cricoid cartilage during the procedure to ensure the weight displayed accurately reflects the amount of their body weight transferred through the patient's cricoid.

Weight data is collected from the time the cricoid operator stands on the scales via a serial data recorder (model SDX, Photologic Ltd., Ottawa, Canada) onto a 2 gigabyte Secure Digital (SD) card. The scales transmit at a rate of 60 data points per second.

#### Aspirate collection

Following successfully placement of the ETT, aspirates from the oropharynx and the trachea will be collected using 12 gauge suction catheters (Unomedical, Sydney, Australia) connected to 40 ml specimen traps (Kendall Argyle™). If no tracheal aspirate can be obtained, 3 ml of normal saline will be instilled into the ETT, the patient ventilated for 5 breaths and the collection repeated. Using a fresh transfer pipette, each aspirate will be transferred to a specimen tube containing 0.5 ml 0.1 M citric acid (pH 2.5) to maintain pepsin stability [42]. The tubes will then be labeled with a unique fibrinogen Plate Number which becomes the means through which all data for the research will be tracked.

#### Pepsin assay

Ufberg's pepsin plate assay is reliant upon test samples containing enzymatically active pepsin. Pepsin activity is highly sensitive to pH with maximum activity between pH 1.5-2.5 and irreversible inactivation occurring above pH 7.5 [43]. Pepsin stored in gastric juice samples at 4°C remains active for up to 3 days (evaluated up to pH 6)[44]. The pH of saliva sampled from patients following episodes of gastric regurgitation has been reported to range from 7-8 [45]. Therefore citric acid (pH 2.5) will be added to aspirates to ensure pepsin activity is maintained prior to testing.

The laboratory will perform the qualitative pepsin plate assay based on fibrinogen digestion according to the method of Ufberg *et al*. [39]. Briefly, 1.25% agarose plates are prepared with bovine fibrinogen (in normal saline) added to a final concentration of 1 mg/ml. Four wells are punched into the agarose, each capable of holding 7 μl of test sample. Porcine pepsin (positive control) and normal saline (negative control) are included on each plate along with a sample of each aspirate from one patient. The plates are left in a humid chamber for up to 24 hours when they are read by visual inspection. A positive result is recorded if there is visible clearing zone around a well. No clearing zone visible after 24 hours is recorded as a negative result. The plate is checked progressively after sample addition initially every 2 hours, in this way a positive tracheal result can be communicated to the clinician.

### Data collection

After the intubation is complete the personnel involved complete a data sheet included in the trial enrollment pack. Data recorded includes patient demographics, procedural details, procedural and post-intubation complications encountered.

*Patient demographic details*; age, date & time of intubation and medical record number (for 28 day follow-up).

*Procedural details*; location of intubation (ED or pre-hospital), lowest pre-intubation GCS, intubation drugs used, experience of intubation operator, evidence and nature of macroscopic aspirate seen and grade of laryngoscopic view obtained.

*Procedural complications*; 3 or more attempts to place the ETT in the trachea, > 10 minutes using conventional laryngoscopy, required change of operator, termination of the attempt at RSI, use of laryngeal mask or surgical airway.

*Post-intubation complications encountered within 30 minutes of RSI*; severe hypotension (Systolic blood pressure < 60 mmHg), severe hypoxaemia (SaO_2 _< 70%), death and cardiac arrest.

The data sheet and SD card containing weight data will be sent to the Trial Coordinator who will enter the data into a password-protected database. Each episode of RSI has a unique randomly assigned number within the database (Plate Number). This number will be linked to the episode of service, and the pepsin assay result will be automatically appended to the corresponding patient data set in the database. The investigators are therefore blinded to the results coming in from the laboratory.

At 28 days the patient notes will be reviewed. The acute physiology and chronic health evaluation (APACHE II) score and diagnostic code will be determined and any treatment for aspiration as an inpatient (discharge diagnosis of aspiration pneumonitis/pneumonia or antibiotic therapy explicitly instituted to treat for aspiration pneumonia) will be noted.

### Sample processing

The aspirates are sent directly to the hospital's pathology laboratory for pepsin detection or, if outside pathology opening hours, stored at 4°C until the laboratory opens. Tamworth and Port Macquarie hospitals have access to a 24 hour pathology laboratory, while Coffs Harbour hospital's pathology service operates between 0700-2400 hours. Each laboratory is blinded to the conduct of CP during the intubation.

### Sample size

In order to adequately power the trial the variation in the rates of aspiration reported in the literature (0.8% to 22%) must be reconciled. Sellick's original data indicates a rate of 11% [2]. Therefore, assuming a rate of 11% aspiration in the control arm, 0% aspiration in the intervention arm and a power of 90% (alpha error = 0.05), 106 patients must be randomised to each group.

### Analysis

Continuous measurements will be expressed with means and standard deviations. Frequencies will be published the variables of interest. Two group comparisons will be undertaken using the Chi-squared test or Fisher's exact test where appropriate. Comparisons of means will utilise an independent *t *test. Ordinal and continuous variables will be compared using a Mann-Whitney test.

### Staff education

All staff assisting with the trial will complete a mandatory education DVD to ensure uniformity can be maintained across the participating hospitals. The DVD consists of an explanation of the trial, reviewing the anatomy of the upper airway, standardising location of the cricoid ring, delivery of 30 - 40 N CP during the intubation, operation of the scales and serial data recorder, and data collection.

## Discussion

The primary aim of this study is two-fold. Firstly, by comparing the incidence of aspiration of pepsin in the intervention and control groups and prospectively documenting the benefits and adverse events associated with 30 - 40 N of cricoid force during RSI, it aims to challenge the notion that CP prevents aspiration. Secondly, since the presence of pepsin in the oropharynx will be indicative of aspiration having occurred prior to intubation by virtue of the injury sustained by the patient, determining the incidence of pre-intubation aspiration will allow us to further evaluate the relevance of CP in the RSI algorithm in ED.

The secondary aim of this study is also two-fold. Firstly, defining any observed correlation between aspiration and pre-intubation Glasgow coma score will provide evidence for indications for the use of CP dependent upon the level of patient consciousness. Secondly, a correlation between detection of pepsin in trachea and development of aspiration syndromes will support the use of Fibrinogen plate assay in a clinical setting for their early detection and treatment.

It is conceivable that this research could also give rise to novel therapy. For example, once the incidence of serious aspiration and a pepsin positive assay is identified, the plate assay for pepsin could potentially be performed at the bedside, enabling doctors to treat aspiration pneumonia much earlier. It may also lead to amendments to the algorithm for RSI if CP is found to be deleterious.

## Trial status

Ongoing.

## List of abbreviations

ANSWER: Amalgamated New South Wales Emergency Research; ANZCTR: Australian New Zealand Clinical Trials Registry; APACHE: Acute Physiology and Chronic Health Evaluation; CP: cricoid pressure; CPR: cardiopulmonary resuscitation; DVD: Digital Versatile Disc; ED: Emergency Department; ETT: Endotracheal Tube; GCS: Glasgow Coma Score; N: newton; NSW: New South Wales; RSI: Rapid Sequence Intubation; SD: Secure Digital.

## Competing interests

The authors declare that they have no competing interests.

### Financial competing interests

None identified.

### Nonfinancial competing interests

None identified.

## Authors' contributions

CT conceived, designed and participated in the co-ordination of the study. JB co-ordinated the study. DC performed the pepsin assay. SD participated in the study design. All authors read and approved the final manuscript.

## Authors' information

CT - Emergency Physician with multiple publications, including two regarding the utility of CP in the Emergency Department.

JB - Research Co-ordinator at the Rural Clinical School, University of Newcastle, Australia.

DC - Clinical Biochemist, Pathology New England.

SD - Emergency Physician, Associate Professor and Director of the Rural Clinical School, University of Newcastle, Australia. Multiple publications.
